# Skin Diseases and Their Treatment

**Published:** 1874-04

**Authors:** Edward B. Gray


Certain Skin Diseases and Their Tteatment.—*By Edward B. Gray, M.D.*—Eczema.—In 1864–5 an undergraduate, aged twenty, consulted me occasionally on account of a moist eczema, shifting about the forehead, but showing a preference for one or other supra-orbital region. I could never find anything wrong in his general health or habits.

His eczema supervened slowly on the subsidence of spasmodic asthma, which had troubled him very frequently from early boyhood. For the first twelve years of his life he had pretty constant nocturnal incontinence of urine. He is now in perfect health, with one exception—that when over-tired, or worried, he gets rather severe clavus-headache on one side.

The sequence of events in this patient's case, read in the light of his family history, makes it, to my mind, in the highest degree probable that this frontal eczema, supervening on asthma, was simply a "transmutation of neurosis" (Trousseau) from his vagus to his trigeminus nerve.

Looking back at my treatment of this case, I fear it was wrong. It was the old-fashioned combination of mild tonics and alteratives. Had the patient's strong neurotic taint been from the first known and kept steadily in view; in other words, had all my efforts been directed to feeding and toning up his nervous system with such remedies as cod-oil and arsenic, his eruption would probably have got well much sooner than it did.

*Acute Syphilitic Lichen* may so closely simulate the primary stage of small-pox eruption as to render the diagnosis at first a matter of difficulty. In 1863, Mary H——, aged twenty-two, came from a neighboring village into the Radcliffe Infirmary, under my care, with moderate swelling and redness of one side of the fauces, and inflammatory enlargement of the glands beneath the corresponding jaw. The throat affection was of three weeks' standing; but she had suffered from general malaise since suppression of the catamenia, three months before. There was nothing in the throat affection, nor in her general condition, to raise any suspicion of syphilis. After three or four days of sharp febrile symptoms, with much pain in back and limbs, an abundant supply of small, hard, red papules appeared, chiefly on face, neck and forearms. Had small-pox been prevailing at the time, few would, I think, have hesitated in pronouncing this to be a case of it, so closely did the eruption and premonitory symptoms resemble those of variola. As, however, there was no smallpox about, careful search was made for some clue to the real nature of the eruption. Some hard and rather enlarged glands were now discovered in one groin. Very soon she began to have severe nocturnal pains in both clavicles. The papules desquamated and cleared away, leaving behind them dark coppery stains, so that, altogether, the syphilitic origin of the eruption seemed beyond reasonable doubt.

*Ringworm* (tinea tonsurans) in the scalp is often hard to cure, and, without very elaborate and repeated microscopic examination, beyond what one can often find time to make, it may be hard to say when it *is* cured. A child has a patch of ringworm on its scalp. You have applied iodine, perchloride of mercury, or some one of the ordinary parasiticides. At the end, say, of a fortnight, the place looks better, but is still somewhat scurfy, and the skin perhaps thicker than natural. How much of this irritation is due simply to the remedies you have been using, and how much to a still vital remnant of the disease, is now and then a hard, and at the same time a pressing, question to settle; as, for instance, when the disease is keeping a boy away from school, greatly to his parents' disgust and impatience. In pale, delicate children I have many times seen the skin of the tinea patch remain thick and scurfy, long after repeated and careful microscopic examination had ceased to find any trace of the parasite. In such cases, if the scalp be allowed entire rest from all local treatment, *washing included*, for a week or so, it will probably have got nearly or quite sound.

